# Prediction aided in vitro analysis of octa-decanoic acid from Cyanobacterium Lyngbya sp. as a proapoptotic factor in eliciting anti-inflammatory properties

**DOI:** 10.6026/97320630013301

**Published:** 2017-09-30

**Authors:** Paramasivan Manivannan, Gangatharan Muralitharan, Nainangu Prasanna Balaji

**Affiliations:** 1 Department of Microbiology, Bharathidasan University, Tiruchirappalli - 620024, Tamilnadu, India;

**Keywords:** Lyngbya, octadecanoic acid, proapoptotic factor, anti-inflammatory activity, MMP-2, Interaction assessment

## Abstract

Marine Lyngbya has been proven as a potent anticancer agent by disrupting microfilament network. Lyngbya and its associated cyanobacterial compounds have been stressed for futuristic advancements in cancer research and have foreseen explicit advancements in the recent era. Moreover, compounds like lyngbyabellins, lyngbyastatins and other derived toxins are significantly studied. Therefore it is of interest to study the efficacies of Lyngbya fatty acid derivatives. Cytotoxicity and DNA laddering studies proves the efficiency and safety of marine Lyngbya. Caspase - 3 along with MMP2 and MMP9 affirms the anti-inflammatory properties. Molecular docking shows that Octadecanoic acid has strong binding affinity to MMP-2. The role of octadecanoic acid as a proapoptotic factor in emancipation of inflammation apart from inducing apoptosis is of interest to study in future.

## Background

The arena of natural products research focuses mainly on Lyngbya owing to the fact of various secondary metabolites [1, 2], especially pertaining to anticancer research. Marine Lyngbya have been a progressive source that has deferred over 260 diversified secondary metabolites contributing a "lion's share" in the field Cyanobacterial metabolites resource (http://www.chem.canterbury.ac.nz/marinlit/marinlit.shtml). Significantly, L.majuscula alone is responsible for nearly 196 different secondary metabolites [3]. The presence of modular non-ribosomal polyketide synthetase (NRPS) and polyketide synthase (PKS) pathways affirms the biotechnological potentials of Lyngbya sp. [4, 5, 6]. Lyngbya has been proven to be an exemplary source in arresting various cancers with varied effects (Table 1). Although marine Lyngbya has been well denoted as a potent therapeutic, molecular dissection of their antiinflammatory attribute needs to be unraveled. Several reviews denote the potentiating nature of marine Lyngbya, nevertheless exploitation of this cyanobacterium as a drug modality remains in development and not yet as a commercial commodity. Moreover, In the Indian context, marine Lyngbya isolated from Gulf of Mannar has not been reported earlier for its antiinflammatory activities. In the present study, marine indigenous Lyngbya from the Gulf of Mannar was assessed for its anti inflammatory along with anti cancer activities. A comparative study to alleviate the extracellular and intracellular metabolites and their efficacies was noted. Furthermore, computational analysis to dissect the mode of action as an anti-inflammatory agent was confirmed through molecular docking to associate the marine Lyngbya's rich natural therapeutic property.

## Methodology

### Sample Collection

**Figure 1:**
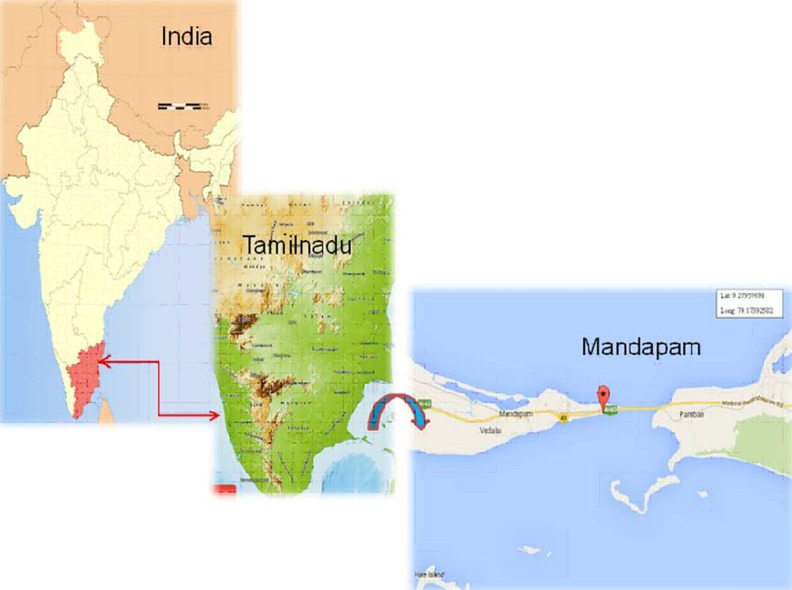
Depicts the sampling site mandapam in the Gulf of Mannar region with accurate eight latitude and longitude.

Cyanobacterial sample was collected from coast of Gulf of Mannar, Rameshwaram, Tamil Nadu, India. The longitude & latitude of location is being identified using GPS locator as N-9° 27 95", E-79° 17 3" (Figure 1). The collected samples were collected as three replicate specimens with distance of 3 m for each sample. The samples observed under the microscope and morphologically characterized.

### Morphological characterization by microscopy 

**Figure 2:**
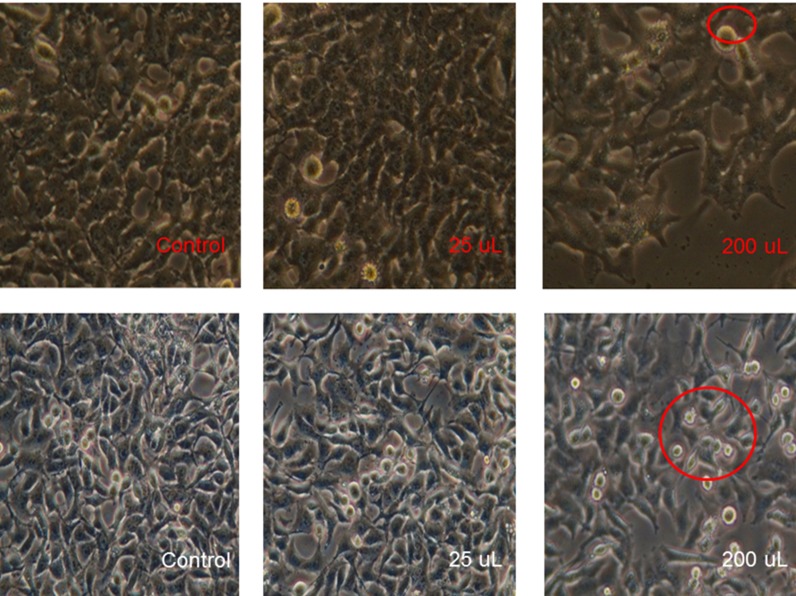
The MTT assay profiles indicating high cytotoxicity of aqueous extracts of 5 Lyngbya sp against HepG2 and MCF cell lines.

The cyanobacterial specimen were observed to be dark green in color, each filaments measures approximately 10 μm in length. They were measured on compound light microscope with a 40x and 10 x ocular lenses with a calibrated optical micrometer. Organism appeared with series of cells surrounded by a tough covering or sheath and mostly long slightly waved. The unbranched filaments are straight, slightly wavy or rarely coiled, and usually form large, layered, leathery mats of varied thickness [7]. The morphology of specimen authenticates for Lyngbya sp (Figure 2).

### Pure culture isolation & mass cultivation

Lyngbya sp isolate were inoculated in MN solid medium (N+) and incubated at 25°C ± 2°C temperature under controlled light intensity for optimizing growth under laboratory conditions. Lyngbya sp was grown in laboratory condition for 12-14 days along with germanium dioxide & cycloheximide to control diatom & green algae contamination. Filaments were picked up selectively and inoculated in to MN liquid media for mass cultivation. Cyanobacterial cultures were initially grown in 250 ml conical flasks MN medium with pH 7.5±0.2 [8], followed with 1000ml MN medium for mass cultivation.

### Extraction of compounds and HPLC Purification

Dried biomass (10 grams) of axenic Lyngbya sp was grounded with solvent mixture of MeOH: H2O (3:1 V/V) using mortar and pestle and stored at 4°C for 24 hr. Extracts were centrifuged at 10,000 rpm for 15 min and supernatant was collected and stored. Aqueous layer was re-extracted with same solvent twice and extracts were concentrated to dryness in rotary evaporator at (55°C), then concentrated material was extracted with 100% methanol and methanol: water (3:1) separately. Extracts were fractioned in Silica Gel column (1.5 x 20 cm) [9] and purified by HPLC system. The active methanolic fraction of column-purified extract was purified further with High performance liquid chromatography. The fraction was detected in reverse phase HPLC ODS-C18 column (4.6mm ID x 25 cm). The mobile phase was mixture of methanol:water of (97:3 to 100 %) added under isocratic conditions. The flow rate was 0.5 ml/min and UV detection performed with 254nm [10, 11].

### GC/MS analysis of compound

The GC- MS was performed on Joel, ACCU TOF GCV. The GC coupled with high-resolution mass spectrometer (HRMS) and with library search uses EI ionization mode. About 20 μl samples were injected at 240°C (Injector temperature) in the column (30 m x0.32 mm ID) with carrier gas as helium. GC oven temperature programme started at 80° C for 2 min followed to final temperature of 280° C. The sample analyzed at constant flow of 2 ml / min. The mass range analyzed at m/z [10 - 600] with ionization detected, recorded using Flame Ionization Detector (FID). The complete Data acquisition was performed by NIST library search.

### DNA extraction

Total genomic DNA extraction was done as described previously [12] and used as a template in PCR reaction. Genomic DNA was extracted from Lyngbya sp by following standard procedure [13]. Eight days old culture was pelleted by centrifugation, then medium was decanted, and the pellet was resuspended in 500 μl of 50 mM Tris-HCL (pH 8.0), 5mM EDTA (pH 8.0), 50 mM Nacl. Lysozyme was added to obtained a final concentration of 1 mg/ml, and the solution was incubated at 55° C for 10 min or until the solution cleared (complete cell lysis). The solution was chilled on ice and extracted with an equal volume of phenol - chloroform-isomylalcohol (25:24:1). Organic extraction was repeated, and the supernatant was added to an equal volume of 4 M ammonium acetate. Total genomic DNA was precipitated by the addition of 2 volumes of isoproponal followed by centrifugation for 10 min at room temperature. Pellet was washed with 70% ethanol, dissolved in TE buffer (10 mMTris- Hcl, 1mM EDTA, and pH 8.0) and stored at -20°C. DNA concentration and purity was measured on a Du 800 Spectrophotometer.

### PCR Amplification

The PCR amplification of DNA with cyanobacterial specific primers was carried out following the protocol of Nubel et al 1997 [14]. The PCR reaction volumes were 50μl containing 1μl of DNA, 1μl of each primer, 25μl of Taq DNA Master Mix and 22μl of H2O. The PCR reactions were performed in an eppendorf Master Cycler gradient as follows; after initial denaturation at 92°C for 2 min 30 cycles of 92°C for 20s, 52°C for 30s and 72°C for 60s. PCR had final extension step at 72°C between 5 to 7min. PCR products were analyzed by electrophoresis in 1.2% agarose gel in 1x TBE buffer stained with ethidium bromide and photographed under UV transilluminator. The sequences of the PCR products were determined by using the BigDye Terminator Cycle Sequencing v2.0 kit on an ABI 310 automatic DNA sequencer (Applied Biosystems, CA, USA).

### Cell lines and Cytotoxicity assays

Cell lines HepG2 and MCF7 were obtained from National Center for Cell Sciences, Pune, India. Cultures were stored in liquid nitrogen, upon usage need they were passaged and thawed for further analysis. The extracts were dissolved in 10% Dimethyl Sulfoxide (DMSO) to give a final concentration of DMSO not more than 0.5% and did not affect cell survival. The viability of cells was assessed by MTT assay using MCF 7 and HepG2 cell lines. The MCF 7 and HepG2 cells were plated separately in 96 well plates at a concentration of 1 x 105 cells/well. After 24 h, cells were washed twice with 100 μl of serum-free medium and starved for an hour at 37°C. After starvation, cells were treated with different concentrations of extract (25-200μg/ml) for 24 h. At the end of the treatment period the medium was aspirated and serum free medium containing MTT (0.5 mg/ml) was added and incubated for 4 h at 37°C in a CO2 incubator. The 50% inhibitory concentration value (IC50) of the crude extracts was identified. The MTT containing medium was then discarded and the cells were washed with PBS (200 μl). The crystals were then dissolved by adding 100 μl of DMSO and this was mixed properly by pipettting up and down. Spectrophotometrical absorbance of the purple blue formazan dye was measured in a microplate reader at 570 nm (BIORAD 680). Cytotoxicity was determined using Graph pad prism5 software.

### DNA Fragmentation

In 24 flat-wells plate, 2x105 MCF 7 and HepG-2 cells (triplicate wells of 105 per well) were incubated with different concentration of sample (25- 200) (105 target cells per well). Fresh DMEM medium was added and incubated for 24 hours, cell sample was collected in 1.5 ml eppendorf tube, spinned down, resuspended with 0.5 ml PBS in 1.5 ml eppendorf tubes, and 55ul of lysis buffer (40 ml of 0.5 M EDTA 5 ml of 1 M TrisCl buffer pH 8.0 5 ml of 100% Triton X-100 50 ml of H2O) was added and kept in ice for 20 min (4°C). After centrifugation in cold at 12,000 g for 30 minutes, the samples were transfered to new 1.5 ml eppendorf tubes and then the supernatant was extracted with 1:1 mixture of phenol: chloroform (gentle agitation for 5 min followed by centrifugation) and precipitated in two equivalence of cold ethanol and one-tenth equivalence of sodium acetate. Then the precipitates were resuspended in 30ul of deionized water-RNase solution (0.4ml water + 5ul of RNase) and 5ul of loading buffer for 30 minutes at 37°C. After agarose gel electrophoresis, the image was observed for DNA shearing in 312nm UV illuminator.

### In vitro activity

#### Western Blotting

Protein expression was determined on the cytosolic extracts from treated cells using western blotting. The cytosolic protein samples were isolated and quantified using Bradford assay and kept at 80°C until use. The protein samples (25μg/well) were electrophoresed by using 10% sodium dodecyl sulfate (SDS)- polyacrylamide gel electrophoresis (PAGE) and transferred to PVDF membrane. Immunoblot analysis was carried out using rabbit anti-rat polyclonal antibody (1:1000) as primary antibody and alkaline phosphatase-conjugated goat anti-rabbit IgG (1:5000) as secondary antibodies, respectively. Target protein was detected using alkaline phosphatase and NBT-BCIP as the substrate. The band intensity was measured densitometrically using Image J software. Briefly, the method consisted of analyzing the integrated densities (the area of the particular band multiplied by the mean value) of individual bands of the scanned blot. β- actin was used to normalize the target and the relative expression was stated as fold ratio.

### Computational studies

#### Active site prediction and preparation of ligands

Ligand binding sites were predicted using Qsite finder (http://www.modelling.leeds.ac.uk/qsitefinder/), surface topology and pocket information were analyzed by theCASTp server (http://stsfw.bioengr.uic.edu/castp/calculation). The results were compared to PDB sum records. The solvent accessible surface area (SASA) was found employing GETAREA (http://curie.utmb.edu/getarea.html). The atomic SASA covered by each cleft was calculated by utilizing radius of water probe 1.4 Å, and the area/energy/residue was calculated. Dielectric constant was set to 80.0, and Poisson-Boltzman method of computation for 20 cycles was used for calculating the electrostatic potential. Ligands namely, rhodopin and octadecanoic acid as obtained by GC-MS analysis was retrieved from the PUBCHEM database (https://pubchem.ncbi.nlm. nih.gov/search/search.cgi) in their canonical SMILES format. Files were then converted into PDB files using openbabel.

### Target receptors and docking analysis

MMP 2 and MMP 9 were chosen for the present analysis. The structures were retrieved from Protein Data Bank (http://www.rcsb.org/pdb) with PDB ID: 1ITV and 1RTG. Solvent accessibility was utilized as a criterion in emancipating the binding nature of the peptides. ASAVIEW [15] was used for Solvent accessibility calculations. The online docking program PATCHDOCK [http://bioinfo3d.cs.tau.ac.il/PatchDock/] was used for rigid docking of the proteins with rhodopin and octadecanoic acid. Ligand interactions were analyzed by Discovery studio (http://accelrys.com/products/discoverystudio/ visualization-download.php) and were viewed with PYMOL.

## Results and Discussion

### Metabolites involved in Bioactivity

**Figure 3:**
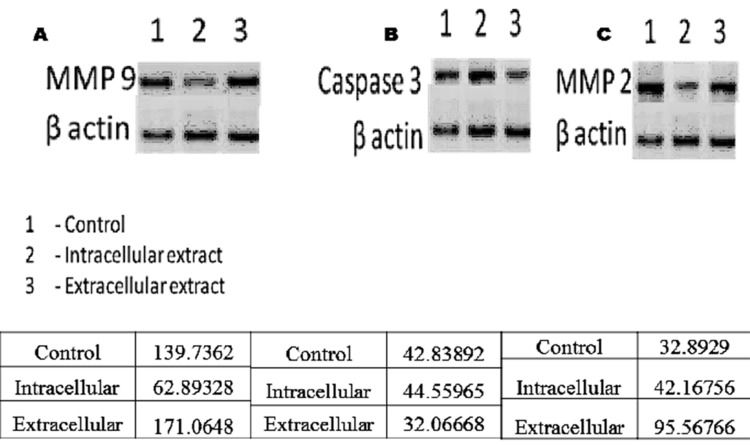
Demonstrates the anti inflammatory profiles of intracellular and extracellular 4 extracts of Lyngbya sp with high fold ratio for extracellular extracts showing its 5 therapeutic profile.

**Figure 4:**
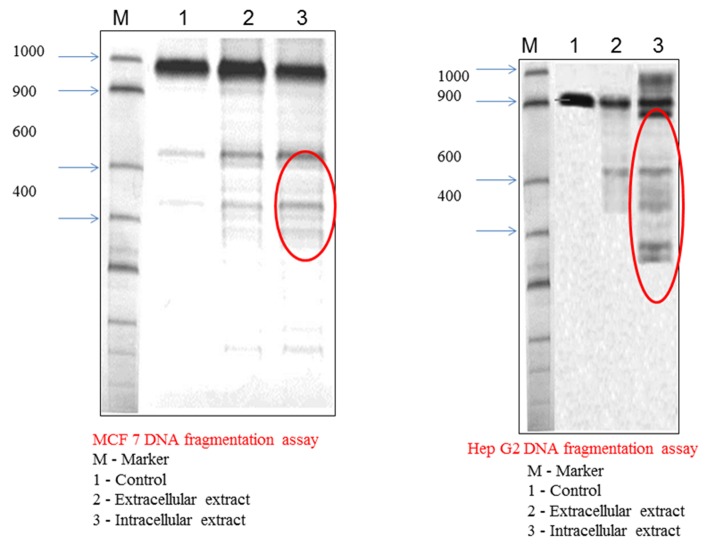
Contrarily shows that intracellular extracts possess high DNA fragmentation 4 activities.

Cyanobacteria continue to be a significant source of compounds that show unprecedented biological activities of pharmaceutical thrust. Cyanobacterial metabolites prove to possess a wide range of biological activities ranging from anti-microbial and immunosuppressant to anti-cancer and anti-HIV. Figure 3 and Figure 4 depict the location map and microscopic images of Lyngbya sp. Surprisingly, Intracellular extract showed significant cytotoxicity and DNA laddering patterns, whereas, extracellular extract illustrates the protein expression differentially in caspases-3, MMP-2 and MMP-9 activities. GC-MS analysis revealed the presence of two principal components namely rhodopin and octadecanoic acid, 3-Hydroxy-2-tetradecyl-methylester (Figure 3). Unsaturated fatty acids have long been proposed for antimicrobial nature. Furthermore, pharmaceutical properties of activities α-linolenic acid are well known [16]. With regard to anticancer activity, linoleic and α-Linolenic acids have been accounted to possess prominent proapoptotic effects and growth inhibitory roles [17]. Although antimicrobial nature of octadecanoic acid has been well documented, their exact role in anticancer and anti inflammatory properties have not been thoroughly studied. The present work is first of its kind in addressing Lyngbya sp. as an anti-inflammatory agent and the key component as octadecanoic acid.

### In vitro activity

200 μL aqueous extracts of Lyngbya sp. show efficient cytotoxicity by MTT assay (Figure 4). The active principles from the aqueous extracts were primarily found to be octadecanoic acid and rhodopin. Previous studies with Terminalia belerica methanolic extracts (TBME) were found to possess phenolics, flavonoids, and other phytochemicals, which render TBME to involve in anticancer activity. Reserpine, tannic acid, quercetin, catechin, and gallic acid/ascorbic acid were found to be active ingredients through HPLC analysis. Cytotoxicity of TBME was demonstrated against A549 and MCF7 cell lines significantly. The cytotoxicity was presumed to be the effector processes of apoptosis, necrosis or cell cycle arrest [18]. Furthermore wildtype p53 upregulation of Bax and downregulation of Bcl-2 that results in apoptosis was categorized as the principle process involved [19, 20]. In the present study, MTT Assay depicts the intracellular extract as the predominant source for cytotoxicity and DNA fragmentation affirms the phenomenon. Till date, there have been ample reports on plants that exhibit both anti-cancer and anti-inflammatory activities. But cyanobacteria in eliciting this response are scarce.

### Western Blotting

**Figure 5:**
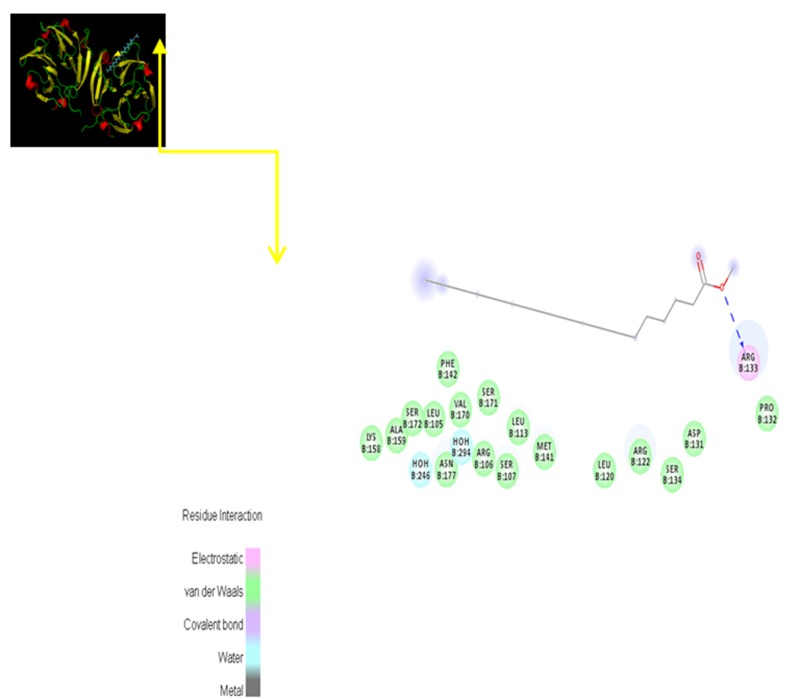
Indicates the hydrogen bond donor ARG133 with varied hydrogen acceptors.

Apoptosis of extracts in general have been regarded as a resultant of Bax/Bcl-2 ratio that leads to cytochrome c release that indeed results in caspase activation to be proceeded as apoptosis through the mitochondrial pathway [21, 22]. Proapoptotic nature of octadecanoic acid might be attributed to cleavage of caspase-3 [23]. Blotting of the treated cell lines revealed the presence of caspase 3 (35 KDa), MMP2 (72 KDa) and MMP9 (92 KDa). The results of blotting clearly indicate the effect of active principles of extracts on the expression profiles of cell lines HepG2 and MCF7 (Figure 5). HepG2 cell line activity can be corroborated with hepatoprotective nature of octadecanoic acid. Free fatty acids exposure over a period of time has implications on reduced glucose stimulated insulin secretion and apoptosis of human pancreatic cells. Proapoptotic effects in this regard were not affirmed through caspases whereas dependent on ceramide pathway and Bcl-2 regulation might possibly have its role [23]. Positive effects of octadecanoic acid methyl ester against breast cancer inhibition might be due to arrest of de novo Diacylglycerol synthesis that induces apoptosis of human breast cancer cells. Activated Phosphokinase C reversed caspase-3 activation might be a modality of inhibition of breast cancer development [24]. Hence octadecanoic acid might have its effect on MMP2 and MMP9 through caspase3 activation and inflammation, leading to apoptotic cell death. Interestingly, extracellular extracts of Lyngbya sp. show high fold percentage of Caspase-3, MMP2 and MMP9 when compared to intracellular extract. DNA fragmentation profiles of the aqueous extracts clearly establish Lyngbya to be a potent anti cancer repertoire. Comparatively, extracellular extracts show increased bioactivity. Hence, the present analysis explains that both intracellular and extracellular extracts as a drug modality in combating inflammatory nature of Lyngbya sp.

### Computational studies

Figure 5 depicts efficient interaction between Octadecanoic acid - MMP2 (-137.63 KJ/mol) than MMP9 (-121.55 KJ/mol). However its efficacy in interaction with caspase3 (-218.89 KJ/mol) is twofold than MMP's indicating high anticancer activities prior to anti inflammatory activities and western blotting results affirm this phenomenon. Hydrophobic interactions between ARG133 render catalytic residues occupied with octadecanoic acid. Similarly, it was earlier proposed that n-hexadecanoic acid in the channel of the enzyme hampered the substrate entry and HIS 48, ASP 49, and a catalytic Ca2+ were found unavailable to the substrate. Further, computational studies showed that nhexadecanoic acid had a binding energy of 58.14 Kcal/mol when compared to the present study with -137.63 KJ/mol. Binding in roller coaster rail model was also seen as in the previous studies [25, 26]. Hence there is a dire need in studying bioactive nature of cyanobacteria and cyanobacterial natural product chemistry still remains underexploited. Nevertheless there have been several limitations and lacunae in addressing bioinformatics potentials for efficient identification and betterment of human health perspective.

## Conclusions

Extracellular extract of Lyngbya sp. show potent antiinflammatory property. Which is evident from their fold percentage in Protein expression showing anti-inflammatory nature by western blotting. Computational results also corroborates by increased binding affinities to Caspase 3, MMP2 and MMP9. HPLC and GC MS confirm the presence of rhodopin and octadecanoic acid as the active principles in eliciting antiinflammatory along with anti-cancer properties. Among which, octadecanoic acid promisingly binds to MMP2 implicating role as a proapoptotic factor. However, further interior research is needed to emancipate octadecanoic acid's role in apoptosis.

## Conflict of interest

The Authors declare that they have no competing interests.

## Figures and Tables

**Table 1:** Notable anticancer compounds from Lyngbya

Compounds	Source	Mode of action
Curacin A	Lyngbya majuscula	Inhibition of microtubule assembly
Dolastatin 15, Cematodin (LU-103793), ILX-651 (Synthadotin)	Lyngbya sp.	Breast cancer abatement
Apratoxin A	Lyngbya sp.	Efficient in arresting early stage adenocarcinoma
Somocystinamide	Lyngbya majuscula/	Cyto toxicity against mouse
A	Schizothrix sp. Assemblage	neuro-2a neuroblastoma
Lyngbic acid and malyngamide C	L. majuscula	Cytotoxic against HT29 colon cancer
Apratoxin D	L. majuscula and L. sordida	Cancer cell toxicity
Apratoxin E,	L. bouillonii	Cytotoxicity
Caylobolide A	L. majuscula	In vitro cytotoxicity against human colon tumor cells
Lyngbyaloside	L. bouillonii	
Lyngbyabellin J, laingolide B, lyngbyapeptin D, lyngbyabellins A and B, lyngbyapeptin A, and lyngbyaloside	L. bouillonii	Cytotoxicity
Curacins B and C	L. bouillonii	Cytotoxicity against murine L-1210 leukemia and human CA46 Burkitt lymphoma cell lines
Itralamide B	L. majuscula	Cytotoxicity against human embryonic kidney (HEK293)
Hantupeptin A	L. majuscula	Cytotoxicity to MOLT-4 leukemia cells and MCF-7 breast cancer cells
Lyngbyabellin A	L. majuscula	Disruption of cellular microfilament network
Lagunamides A and B	L. majuscula	Cytotoxic activity against P388 murine leukemia cell lines

## Abbreviations

DNA: Deoxyribonucleic acid
MMP: Matrix metalloproteinase
HPLC: High performance Liquid Chromatography
GC-MS: Gas Chromatography Mass Spectrophotometry
MTT: (3-[4, 5-dimethylthiazol-2-yl]-2,5 diphenyl tetrazolium bromide), 5-bromo-4-chloro-3-indolyl-phosphate
NBT: nitro blue tetrazolium
